# Final analysis of a phase II study of nivolumab in combination with ipilimumab for unresectable chemotherapy‐naive advanced melanoma

**DOI:** 10.1111/1346-8138.15514

**Published:** 2020-08-18

**Authors:** Kenjiro Namikawa, Yoshio Kiyohara, Tatsuya Takenouchi, Hisashi Uhara, Hiroshi Uchi, Shusuke Yoshikawa, Sumiko Takatsuka, Hiroshi Koga, Naoko Wada, Hironobu Minami, Masahiro Hatsumichi, Yoshinobu Namba, Naoya Yamazaki

**Affiliations:** ^1^ Department of Dermatologic Oncology National Cancer Center Hospital Tokyo Japan; ^2^ Dermatology Division Shizuoka Cancer Center Hospital Shizuoka Japan; ^3^ Department of Dermatology Niigata Cancer Center Hospital Niigata Japan; ^4^ Department of Dermatology Shinshu University School of Medicine Matsumoto Japan; ^5^ Department of Dermatology Graduate School of Medical Sciences Kyushu University Fukuoka Japan; ^6^ Department of Medical Oncology/Hematology Kobe University Graduate School of Medicine Kobe Japan; ^7^ Ono Pharmaceutical Co., Ltd. Osaka Japan; ^8^ Present address: Department of Dermatology Sapporo Medical University Sapporo Japan

**Keywords:** ipilimumab, Japan, melanoma, mucosal, nivolumab

## Abstract

Nivolumab plus ipilimumab combination is currently one of the preferred regimens for advanced melanoma in recently updated clinical practice guidelines. However, the evidence on the efficacy of the combination for acral or mucosal subtypes remains less robust. This is the final analysis of a multicenter, open‐label, uncontrolled phase II study that investigated the long‐term efficacy and safety in treatment‐naive Japanese patients with advanced melanoma, including acral or mucosal subtypes, and subsequent therapy after discontinuation of the investigational agents. Patients received four doses of nivolumab (1 mg/kg i.v.) in combination with ipilimumab (3 mg/kg i.v.) at 3‐week intervals, followed by doses of nivolumab (3 mg/kg i.v.) at 2‐week intervals. The median follow‐up period was 20.8 months (range, 5.2–35.0). The centrally and locally assessed objective response rates were both 43.3% (13/30; 95% confidence interval [CI], 25.5–62.6). Median progression‐free survival was not reached (95% CI, 3.02–not reached), and median overall survival was also not reached (95% CI, 19.52–not reached). The 30‐month progression‐free survival and overall survival rates were 50.3% and 54.2%, respectively. No new safety concerns were detected. After discontinuation of the investigational agents, 83.3% of patients received some form of subsequent therapy including 43.3% of patients who received nivolumab monotherapy and 26.7% of patients who received radiotherapy. Of the four patients who discontinued the investigational agents because of immune‐related adverse events, two received subsequent therapy (nivolumab and ipilimumab, respectively) and the other two showed long‐term treatment‐free survival (659 and 590 days, respectively). Long‐term survival with nivolumab plus ipilimumab was observed in Japanese patients with melanoma including acral and mucosal subtypes, which is consistent with the CheckMate 067 study. Many patients continued to receive some form of treatment safely after stopping treatment with nivolumab plus ipilimumab.

## Introduction

Treatment of advanced‐stage melanoma involves the use of agents that target immune checkpoint proteins such as programmed death 1 (PD‐1) and cytotoxic T‐lymphocyte‐associated antigen 4 (CTLA‐4). Ipilimumab is an antibody to CTLA‐4 and is hypothesized to have a synergistic effect in combination with nivolumab, an antibody to PD‐1, based on the mechanism of action of anti‐CTLA‐4 and anti‐PD‐1 checkpoint blockade.^1^, ^2^, ^3^ The survival benefit of nivolumab plus ipilimumab has been shown in clinical studies of patients with advanced melanoma,^4^, ^5^, ^6^, ^7^ advanced renal‐cell carcinoma,^8^ and non‐small‐cell lung cancer.^9^ Currently, nivolumab plus ipilimumab combination is considered to be one of the preferred regimens as the first‐line systemic therapy for advanced melanoma in recently updated clinical practice guidelines for melanoma.^10^, ^11^, ^12^

Melanoma has several clinically and pathologically distinguishable subtypes: cutaneous, mucosal, uveal and unknown primary melanomas. Cutaneous melanomas are further categorized into superficial spreading melanoma, nodular melanoma, lentigo maligna melanoma and acral melanoma. In Japan, the annual incidence of invasive melanoma is 1.7 (2.2 if *in situ* or stage‐unknown melanomas are included) per 100 000 person‐years, and the acral and mucosal subtypes are common.^13^, ^14^, ^15^ Acral and mucosal subtypes rarely harbor *BRAF* mutation,^16^, ^17^ and several previously published studies suggested that immune checkpoint inhibitors may be less efficacious for acral or mucosal subtypes than for non‐acral cutaneous melanoma.^18^, ^19^, ^20^ However, the evidence on the efficacy of nivolumab plus ipilimumab combination for these melanoma subtypes remains less robust, which is partly owing to lower incidences of these subtypes in Caucasians.

Long‐term efficacy and safety of nivolumab plus ipilimumab were recently reported in a randomized, double‐blind, phase III study (CheckMate 067 study), in which nivolumab plus ipilimumab or nivolumab alone was compared with ipilimumab alone in patients with metastatic melanoma.^21^ At a minimum follow up of 60 months, the median overall survival was more than 60.0 months (median not reached) in the nivolumab plus ipilimumab group and 36.9 months in the nivolumab alone group, as compared with 19.9 months in the ipilimumab alone group. In the CheckMate 067 study, the outcomes after discontinuation of investigational agents were also analyzed, and 46% of patients received subsequent therapy after nivolumab plus ipilimumab combination therapy. Of note, the CheckMate 067 study was conducted outside Japan. An open‐label, single‐arm, multicenter phase II (ONO‐4538‐17) study of Japanese treatment‐naive advanced melanoma patients demonstrated the clinical efficacy and safety of nivolumab plus ipilimumab.^22^ However, neither long‐term survival and safety data nor treatment patterns after discontinuation of these investigational agents have been reported. Therefore, this final analysis of the ONO‐4538‐17 study aimed to investigate the long‐term efficacy and safety of nivolumab plus ipilimumab, and subsequent therapy after stopping nivolumab plus ipilimumab in Japanese treatment‐naive advanced melanoma patients including those with acral and mucosal subtypes. Additionally, we also tried to explore the difference in efficacy of nivolumab plus ipilimumab according to the primary tumor sites within the acral subtype (e.g. subungual vs palmoplantar site) or within the mucosal subtype (e.g. nasal cavity vs oral cavity vs rectum).

## Methods

### Study design

The phase II (ONO‐4538‐17) study was a multicenter, open‐label, uncontrolled study. The study design was described previously.^22^ This final analysis was conducted in compliance with the International Ethical Guidelines for Biomedical Research Involving Human Subjects, Good Clinical Practice guidelines, the Declaration of Helsinki and local laws. All patients provided written informed consent. This study was approved by the relevant institutional review boards or independent ethics committee at each institution. The study was registered at JAPIC‐CTI under the identifier no. 152869.

### Treatments

Patients received four doses of nivolumab (1 mg/kg i.v.) in combination with ipilimumab (3 mg/kg i.v.) at 3‐week intervals, followed by doses of nivolumab (3 mg/kg i.v.) at 2‐week intervals. For patients who continued administration of nivolumab in combination with ipilimumab after the marketing approval of nivolumab was announced on May 2018, commercially available drugs were administrated. Each cycle of the study treatment lasted 6 weeks. The study treatment was continued until progressive disease was diagnosed by the investigator or sub‐investigator according to the Response Evaluation Criteria in Solid Tumors (RECIST) guidelines, version 1.1.

The following therapies were prohibited during the study period: immunosuppressants; corticosteroids at a prednisolone equivalent dose of more than 10 mg/day; antimalignant tumor agents (e.g. chemotherapy, molecular‐targeted therapy or immunotherapy); surgical therapy for malignant tumor; radiotherapy; radiopharmaceuticals (except if used for tests and diagnosis); bisphosphonate products and anti‐receptor activator of nuclear factor‐κB ligand antibody products (except if they were continued from study enrollment using the same dosing regimen); transplant therapy; and all other investigational agents.

Study treatment was discontinued in the event of a dose interruption lasting more than 6 weeks, except for dose interruptions for prolonged steroid tapering to manage drug‐related adverse events (AE).

### Patients

Eligible patients were those who met all of the following criteria at the time of enrollment: men or women aged 20 years or older at the time of informed consent; patients with histologically or cytologically confirmed malignant melanoma; patients diagnosed with unresectable stage III/IV or recurrent malignant melanoma according to the International Union Against Cancer TNM Classification of Malignant Tumors (seventh edition); patients with one or more measurable lesions defined by the RECIST guidelines, version 1.1, on diagnostic imaging within 14 days prior to enrollment in the study (for patients who had undergone radiotherapy for the measurable lesion, disease progression must have been confirmed on diagnostic imaging after radiotherapy); patients with no history of treatment with systemic antimalignant tumor agents (e.g. chemotherapy, molecular‐targeted therapy or immunotherapy) for malignant melanoma; patients with an Eastern Cooperative Oncology Group performance status of 0 to 1; and patients with a life expectancy of more than 90 days. Details of the exclusion criteria have been previously reported.^22^

### Assessments

#### Efficacy

The primary end‐point was centrally assessed objective response rate (ORR). Secondary end‐points were locally assessed ORR, median progression‐free survival (PFS) and overall survival (OS), PFS and OS rates, and best overall response (BOR), including complete response, partial response and stable disease.

Progression‐free survival was calculated as follows: PFS (day) = day of overall response of progressive disease or day of all‐cause death, whichever occurred first − day of starting the study treatment + 1. OS was calculated as follows: OS (day) = day of all‐cause death − day of starting the study treatment + 1. Efficacy by tumor type was assessed by evaluating changes in tumor diameter over time in patients according to the RECIST guidelines, version 1.1.

#### Safety

The frequencies of AE, treatment‐related AE and immune‐related AE (irAE) were evaluated for safety during the treatment phase and up to 100 days after the last dose of the investigational agents. AE severity was graded according to the National Cancer Institute Common Terminology Criteria for Adverse Events, version 4.0, Japan Clinical Oncology Group Version.

#### Subsequent therapy

The information on the first subsequent therapy after meeting the specific discontinuation criteria of the investigational agents was collected. Collection of information on the second or more subsequent therapies was optional. Treatment after discontinuation of the investigational agents, including nivolumab monotherapy, was considered a subsequent therapy.

### Statistical analysis

The safety set included all enrolled patients who received nivolumab or ipilimumab at least once. Efficacy was evaluated in the full analysis set, defined as patients in the safety set with evaluable efficacy data.

Descriptive statistics were used for baseline demographic and clinical characteristics, with *n* (%) for categorical variables and median (range) for continuous variables. PFS and OS were evaluated using Kaplan–Meier analysis. Changes from baseline in tumor diameter were plotted against time. SAS version 9.3 (SAS Institute, Cary, NC, USA) was used for the statistical analysis.

## Results

### Patients

All of the 30 patients enrolled in the phase II (ONO‐4538‐17) study were evaluated in this final analysis. The data cut‐off date was 17 July 2018 and the median follow‐up period was 20.8 months (range, 5.2–35.0). The baseline demographic and clinical characteristics of the patients have been described previously.^22^

### Efficacy

**Figure 1:**
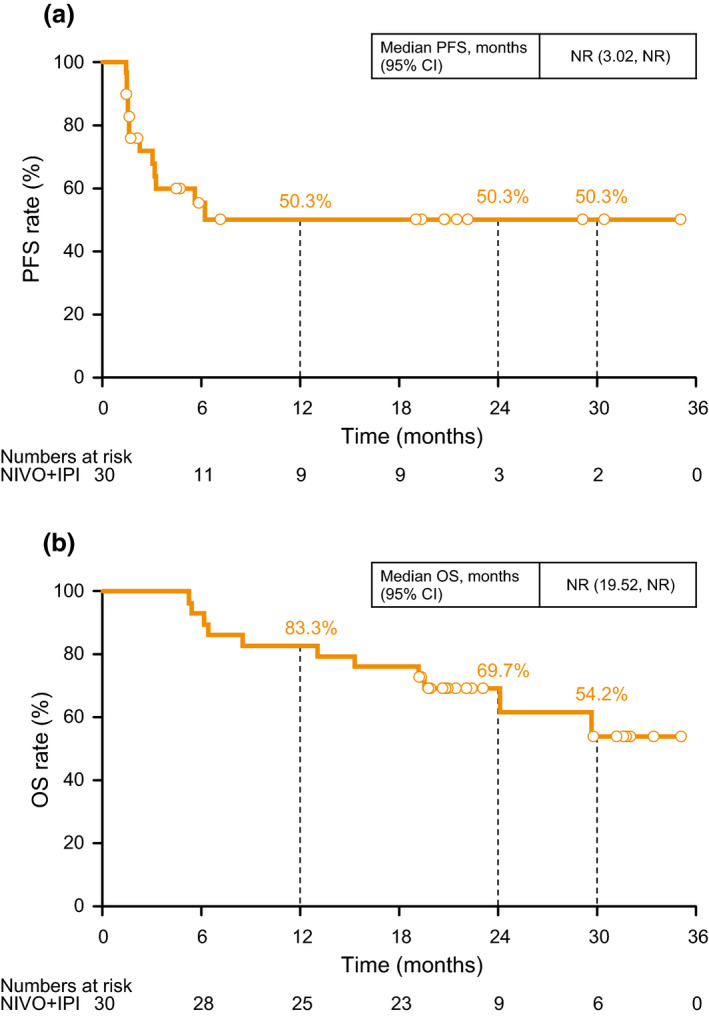
(a) Progression‐free survival (PFS) and (b) overall survival (OS). CI, confidence interval; IPI, ipilimumab; NIVO, nivolumab; NR, not reached.

**Figure 2:**
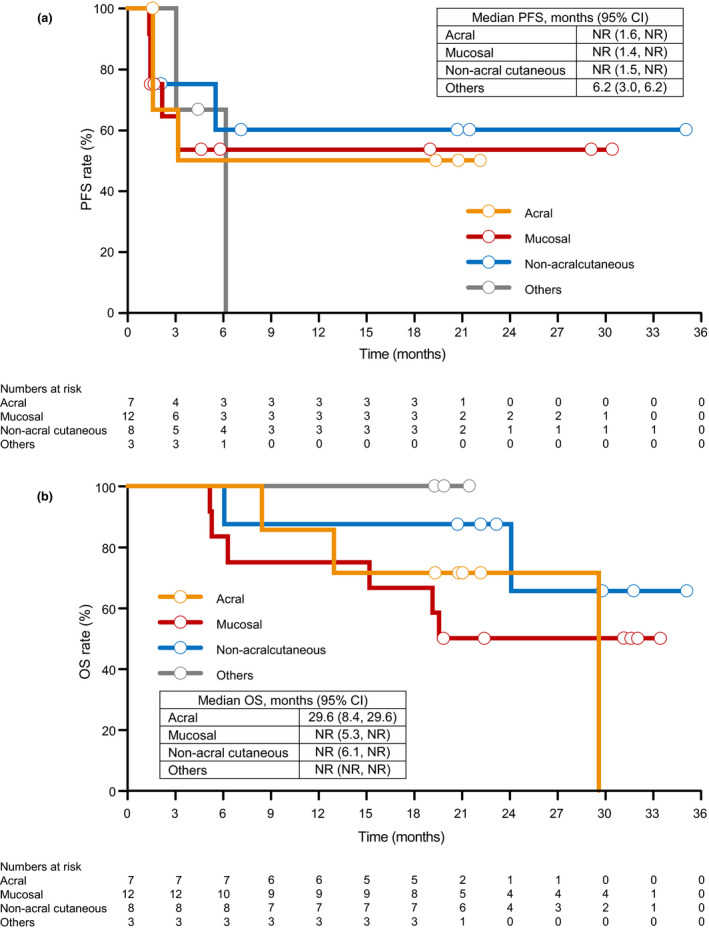
(a) Progression‐free survival (PFS) and (b) overall survival (OS) by tumor subtype. Others include ocular subtype and unknown. CI, confidence interval; NR, not reached.

The centrally and locally assessed ORR were not changed from those in the original report (both 43.3%, 13/30; 95% confidence interval [CI], 25.5–62.6). In the final analysis, the median PFS was not reached (NR) (95% CI, 3.02–NR) and the median OS was also NR (95% CI, 19.52–NR). The 30‐month (2.5‐year) PFS and OS rates were 50.3% and 54.2%, respectively (Fig. 1). PFS and OS by tumor subtype are shown in Figure 2.

**Figure 3:**
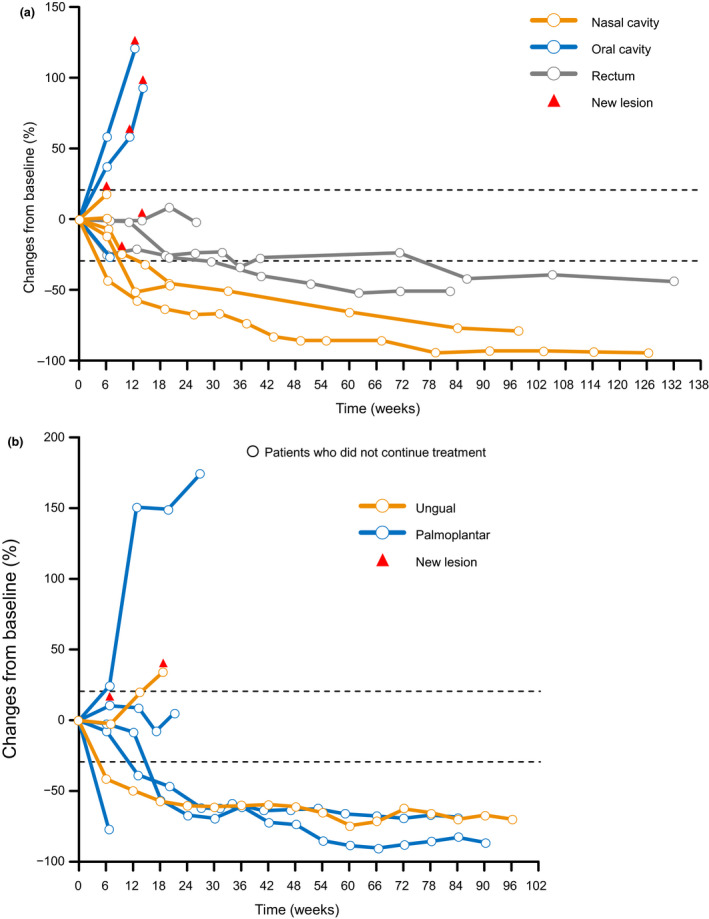
Changes in tumor diameter over time by (a) mucosal site and (b) acral site.

Changes in tumor diameter in individual patients with the mucosal subtype are shown in Figure 3(a). A reduction in tumor diameter was seen in six of 11 patients with mucosal subtype (tumor diameter was not evaluated in one patient). According to the mucosal site, five, three and three patients had the tumor located in the nasal cavity, oral cavity and rectum, respectively. Of these, tumor shrinkage was observed in three, one and two patients, respectively.

**Figure 4:**
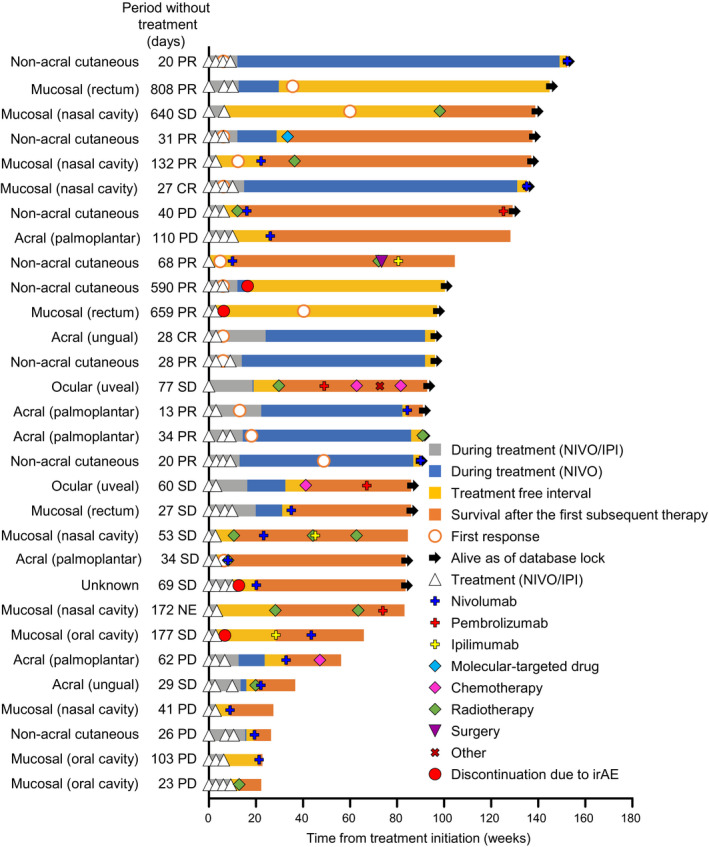
Treatment pattern and course in each patient. In this figure, complete response (CR), partial response (PR), stable disease (SD) or progressive disease (PD) represent the best overall response in each patient. IPI, ipilimumab; irAE, immune‐related adverse event; NE, not evaluable; NIVO, nivolumab.

Changes in tumor diameter in individual patients with the acral subtype are shown in Figure 3(b). A reduction in tumor diameter was seen in five of seven patients with the acral subtype. In the two patients with ungual melanoma, BOR was complete response in one patient and stable disease in the other (Figs 3b,4).

### Safety

AEs and treatment‐related AEs are shown in Table 1. Of the 30 patients, 10 discontinued treatment because of treatment‐related AE. Of the 10 discontinuations, four patients discontinued treatment due to irAE. All patients with these irAE had recovered or were recovering.

**Table 1:** Adverse events and treatment‐related adverse events occurring in three or more patients

Safety analysis set *n* = 30	Adverse events		Treatment‐related adverse events	
Event	All grades	Grades III–IV	All grades	Grades III–IV
Any events	30 (100.0)	23 (76.7)	30 (100.0)	23 (76.7)
Rash	18 (60.0)	2 (6.7)	18 (60.0)	2 (6.7)
Diarrhea	17 (56.7)	1 (3.3)	17 (56.7)	1 (3.3)
Pyrexia	15 (50.0)	1 (3.3)	14 (46.7)	1 (3.3)
Lipase increased	12 (40.0)	7 (23.3)	12 (40.0)	7 (23.3)
Alanine aminotransferase increased	11 (36.7)	3 (10.0)	11 (36.7)	3 (10.0)
Aspartate aminotransferase increased	11 (36.7)	2 (6.7)	11 (36.7)	2 (6.7)
Pruritus	10 (33.3)	0	10 (33.3)	0
Decreased appetite	9 (30.0)	1 (3.3)	8 (26.7)	1 (3.3)
Hepatic function abnormal	7 (23.3)	4 (13.3)	7 (23.3)	4 (13.3)
Malaise	7 (23.3)	1 (3.3)	7 (23.3)	1 (3.3)
Hypothyroidism	7 (23.3)	0	7 (23.3)	0
Hyponatremia	6 (20.0)	5 (16.7)	5 (16.7)	4 (13.3)
Vomiting	6 (20.0)	1 (3.3)	6 (20.0)	1 (3.3)
Constipation	6 (20.0)	1 (3.3)	5 (16.7)	1 (3.3)
Headache	6 (20.0)	1 (3.3)	5 (16.7)	1 (3.3)
γ‐Glutamyltransferase increased	5 (16.7)	3 (10.0)	5 (16.7)	3 (10.0)
Amylase increased	5 (16.7)	1 (3.3)	5 (16.7)	1 (3.3)
Arthralgia	5 (16.7)	0	5 (16.7)	0
Fatigue	5 (16.7)	0	5 (16.7)	0
Stomatitis	5 (16.7)	0	3 (10.0)	0
Viral upper respiratory tract infection	5 (16.7)	0	0	0
Rash maculo‐papular	4 (13.3)	1 (3.3)	4 (13.3)	1 (3.3)
Nausea	4 (13.3)	0	4 (13.3)	0
Blood alkaline phosphatase increased	4 (13.3)	0	4 (13.3)	0
Diabetes mellitus	3 (10.0)	2 (6.7)	1 (3.3)	1 (3.3)
Hypoalbuminemia	3 (10.0)	1 (3.3)	2 (6.7)	1 (3.3)
Upper respiratory tract infection	3 (10.0)	1 (3.3)	1 (3.3)	0
Vitiligo	3 (10.0)	0	2 (6.7)	0
Dysgeusia	3 (10.0)	0	2 (6.7)	0
Anemia	3 (10.0)	0	2 (6.7)	0

Data are presented as *n* (%).

### Treatment pattern in each patient and subsequent therapy

The treatment course in each patient is shown in Figure 4. At the end of the follow‐up period, 19 of 30 patients were alive, 12 of 13 patients with at least partial response were alive, and six of nine patients with stable disease were alive. All patients discontinued study treatment before the final analysis.

**Figure 5:**
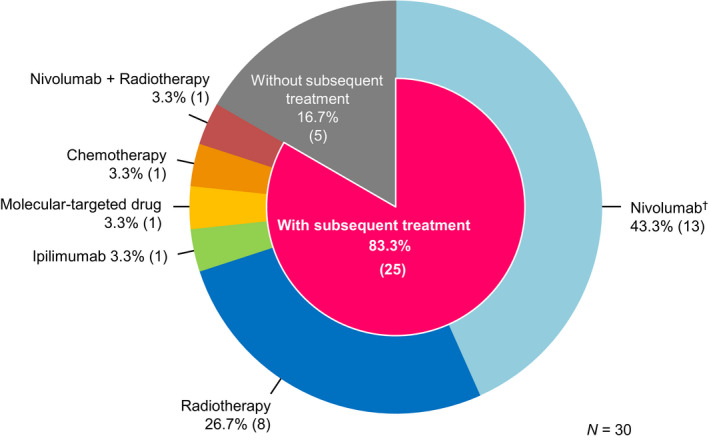
Subsequent therapy just after investigational agent discontinuation. ^†^Four of 13 patients continued administration of nivolumab as a commercially available drug after acquisition of marketing approval.

Subsequent therapy after discontinuation is shown in Figure 5. Of the 30 patients in this follow‐up study, 83.3% (*n* = 25) of patients in the discontinuation group received some form of subsequent therapy. The breakdown of the first subsequent therapy was nivolumab in 43.3% of patients (*n* = 13) and radiotherapy in 26.7% of patients (*n* = 8). Four of 13 patients continued administration of nivolumab as a commercially available drug after acquisition of marketing approval.

Of the four patients who discontinued the investigational agents because of irAE, two patients discontinued due to diarrhea (grade 3) and interstitial lung disease (grade 2), respectively. These patients did not receive a subsequent therapy. The BOR in these two patients was partial response (Fig. 4); one patient with the non‐acral cutaneous melanoma had an initial response during treatment with the investigational agents, but the other patient with the mucosal subtype (site: rectum) did not. The latter patient remained treatment‐free after discontinuation and had an initial response while the patient was without treatment. This patient was alive for 659 days without treatment at the end of the study. The other two patients who discontinued had stable disease at the end of the study. Of these, one patient with an unknown primary tumor discontinued treatment due to enteritis (grade 3), and resumed nivolumab after a 69‐day washout of the investigational agents. The irAE was managed with symptomatic treatment including an antiflatulent but not with steroid treatment. This patient was alive at the end of the study. One patient with the mucosal subtype (site: oral cavity) discontinued treatment for 177 days due to interstitial lung disease (grade 2), and resumed ipilimumab followed by nivolumab. For the management of irAE in this patient, prednisolone was administrated. This patient died by the end of the study.

## Discussion

The present study investigated the long‐term efficacy of nivolumab plus ipilimumab in Japanese melanoma patients, especially those with acral or mucosal subtypes, the relationship between real‐world treatment patterns and prognosis, and safety of this combination therapy. In this final analysis, the ORR and the 30‐month (2.5‐year) PFS and OS rates (43.3%, 50.3%, and 54.2%, respectively) were comparable to those of the CheckMate 067 study,^6^, ^21^ which did not include Japanese patients. In addition, the median OS was NR. Considering these results and the 5‐year OS (52%) in the CheckMate 067 study, nivolumab plus ipilimumab combination therapy is expected to improve the long‐term survival of Japanese patients with advanced melanoma. The safety profile of nivolumab plus ipilimumab combination therapy was consistent with that reported in previous studies.^5^, ^6^, ^7^ No new safety concerns were detected during this final analysis.

Several studies have reported poor survival in acral and mucosal subtypes,^23^, ^24^, ^25^, ^26^ which are more common in Japanese patients compared with Caucasians. Therefore, there is a need to find effective treatments for these rarer melanoma subtypes. In a multicenter, prospective phase II study of nivolumab monotherapy for mucosal melanoma, the ORR and 1‐year OS rate were 23.5% and 50.0%, respectively.^27^ In the ONO‐4538‐17 study, the ORR and 1‐year OS rate in patients with mucosal melanoma were 33.3%^22^ and 75.0% (data not shown), respectively. Thus, the outcomes of treatment with nivolumab plus ipilimumab in Japanese patients with the mucosal subtype in the present study appear to be more favorable than those of treatment with nivolumab alone in similar patients.^27^ A reduction in tumor diameter was observed in the various mucosal sites: three of five patients with tumors in the nasal cavity, two of three patients with tumors in the rectum and one of three patients with tumors in the oral cavity (Fig. 3a). However, because of the small sample size in this study, further studies are needed to clarify the difference in sensitivity to nivolumab plus ipilimumab by site in patients with the mucosal subtype.

The efficacy of anti‐PD‐1 antibody monotherapy in patients with acral melanoma, particularly in those with ungual melanoma, was found to be poor in the JAMP study (ORR was 8.6%).^28^ In the present study, only two patients had ungual melanoma: one had complete response and one had stable disease (Figs 3b,4). This suggests that nivolumab plus ipilimumab combination therapy may be a favorable option in patients with acral melanoma (ungual site); however, more evidence from a larger sample size is needed.

Regarding subsequent therapy, 13 of 30 (43.3%) patients received nivolumab and eight of 30 (26.7%) patients received radiotherapy. In this study, the investigational agent was discontinued in the event of a dose interruption lasting more than 6 weeks for any reason, except for dose interruptions for prolonged steroid tapering to manage drug‐related AE. Furthermore, four patients discontinued nivolumab due to termination of the investigational agent provision, as a result of approval acquisition, and continued nivolumab as a commercially available drug. Therefore, a possible reason for the high rate of nivolumab as a subsequent therapy is that clinically one series of nivolumab treatment was counted as a subsequent therapy in this study.

In the present study, four patients discontinued study treatment due to irAE, two of whom showed long‐term treatment‐free survival (659 and 590 days when follow up was stopped). Although the sample size was small, these findings are consistent with those of the CheckMate 067 study, in which the long‐term OS of patients who stopped nivolumab plus ipilimumab did not differ from that of patients who continued treatment.^29^, ^30^ The other two patients, who discontinued due to irAE, received nivolumab or ipilimumab monotherapy as a subsequent therapy. This observation suggests that some patients can safely resume treatment with nivolumab or ipilimumab alone even after experiencing an irAE (with nivolumab plus ipilimumab) through appropriate irAE management.

The present study has some limitations. These include the lack of a comparator group, the open‐label design and the small sample size.

In conclusion, long‐term survival with nivolumab plus ipilimumab was confirmed in Japanese patients, including those with acral and mucosal melanoma. No new safety concerns were reported. Many patients continued to receive some form of treatment safely after stopping combination treatment with nivolumab plus ipilimumab. Differences in efficacy according to the primary tumor sites within acral subtypes or within mucosal subtypes, which have been grouped together in this study, may be worth considering for further research.

## Conflict of Interest

N. Y., Y. K. and H. Uhara are members of the Nivolumab/Ipilimumab Appropriate Use Committee for Melanoma, which is sponsored by Ono Pharmaceutical and Bristol Myers Squibb. K. N. received research funding from Ono Pharmaceutical and personal fees from Ono Pharmaceutical, Bristol Myers Squibb, MSD, Novartis Pharma, Toray Industries and Takara Bio. Y. K. received research funding from Ono Pharmaceutical and Bristol Myers Squibb; and speaker’s fees, conference registration fees and/or travel or accommodation expenses from Ono Pharmaceutical, Bristol Myers Squibb and Chugai Pharmaceutical. T. T. received research funding from Ono Pharmaceutical. H. Uhara received research funding from MSD, Ono Pharmaceutical, Bristol Myers Squibb, Chugai Pharmaceutical, Novartis Pharma, Takara Bio and Kyowa Kirin; consultancy or commission fees from MSD, Ono Pharmaceutical, Bristol Myers Squibb, Chugai Pharmaceutical, Novartis Pharma and Roche Diagnostics; a fellowship and/or research or education grants from Ono Pharmaceutical and Mochida Pharmaceutical; and speaker’s fees from MSD, Ono Pharmaceutical, Bristol Myers Squibb, Chugai Pharmaceutical and Novartis Pharma. H. Uchi does not have any conflicts of interest to declare. S. Y. received research funding from Ono Pharmaceutical. S. T. received research funding from Ono Pharmaceutical. H. K. does not have any conflicts of interest to declare. N. W. received research funding from Bristol Myers Squibb and Ono Pharmaceutical. H. M. received research funding from Bristol Myers Squibb, Ono Pharmaceutical, Asahi‐Kasei Pharma, Astellas, AstraZeneca, Bayer, Boehringer Ingelheim, Chugai Pharmaceutical, Daiichi‐Sankyo, Sumitomo Dainippon Pharma, Eisai, Kyowa Kirin, Eli Lilly, Merck Serono, MSD, Nihon Shinyaku, Pfizer, Sanofi, Taiho, Takeda, Teijin Pharma, Yakult, CSL Behring, Nihon Kayaku, Shionogi and Novartis Pharma; personal fees from Bristol Myers Squibb, Ono Pharmaceuticals, Bayer, Boehringer Ingelheim, Celgene, Chugai, Daiichi‐Sankyo, Sumitomo Dainippon Pharma, Eisai, Janssen, Eli Lilly, Merck Serono, MSD, Otsuka Pharmaceutical, Pfizer, Sanofi, Shire, Taiho, Takeda, Genomic Health, Novartis Pharma and Abbie; and other supports from AstraZeneca, Bayer, Bristol Myers Squibb, Chugai Pharmaceutical, MSD, Ono Pharmaceutical, Pfizer, Taiho and Novartis Pharma. M. H. and Y. N. are employees of Ono Pharmaceutical. N. Y. received research funding, speaker’s fees, conference registration fees and/or travel or accommodation expenses from Bristol Myers Squibb, Ono Pharmaceutical, MSD and Novartis Pharma.
